# IB-MAC: Transmission Latency-Aware MAC for Electro-Magnetic Intra-Body Communications

**DOI:** 10.3390/s19020341

**Published:** 2019-01-16

**Authors:** Seungmin Kim, JeongGil Ko

**Affiliations:** Department of Computer Engineering, Ajou University, Suwon 16499, Korea; dhddlehd@ajou.ac.kr

**Keywords:** wireless body area networks, intra-body communications, MAC layer protocol

## Abstract

Intra-body Communication (IBC) is a communication method using the human body as a communication medium, in which body-attached devices exchange electro-magnetic (EM) wave signals with each other. The fact that our human body consists of water and electrolytes allows such communication methods to be possible. Such a communication technology can be used to design novel body area networks that are secure and resilient towards external radio interference. While being an attractive technology for enabling new applications for human body-centered ubiquitous applications, network protocols for IBC systems is yet under-explored. The IEEE 802.15.6 standards present physical and medium access control (MAC) layer protocols for IBC, but, due to many simplifications, we find that its MAC protocol is limited in providing an environment to enable high data rate applications. This work, based on empirical EM wave propagation measurements made for the human body communication channel, presents IB-MAC, a centralized Time-division multiple access (TDMA) protocol that takes in consideration the transmission latency the body channel induces. Our results, in which we use an event-based simulator to compare the performance of IB-MAC with two different IEEE 802.15.6 standard-compliant MAC protocols and a state-of-the art TDMA-based MAC protocol for IBC, suggest that IB-MAC is suitable for supporting high data rate applications with comparable radio duty cycle and latency performance.

## 1. Introduction

Intra-body communication (IBC) is a communication technology in which the devices attached on the human body exchange electro-magnetic (EM) waves through the human body itself as a communication medium to exchange messages with each other. The concept of IBC was introduced by Zimmerman [1] as a way to create a personal area network (PAN), and the idea was that a human body consists of water and electrolytes, so the EM waves could be effectively transferred through the body to exchange data. With increasing numbers (and the distribution) of wearable devices, there is a need to increase mechanisms for managing a network around the human body while maintaining low power-usage profiles. While technologies such as Bluetooth Low Energy (BLE) are widely being used in current day devices, the increase in the quantity of such devices can saturate the wireless channel since these devices operate on the 2.4 GHz Industry-Science-Medical (ISM) band, in which many commercial wireless standards operate on. Furthermore, in mission sensitive applications, such as military or clinical applications, the interference induced by external factors can degrade the applications’ performance and usability in general. We find the use of IBC in such applications to be very useful. By using the human body as the communication medium, EM waves can simply flow in the human body (as if we were using a wired communication mechanism) and the signals that we send through this interface can be more tolerant towards external interference. In addition to this, given that the signals are kept within the body, chances of the signal being eavesdropped can reduce as well.

Given such potential benefits, the IEEE, using the 802.15.6 standards, defined a set of physical (PHY) and medium access control (MAC) layer standards to support such IBC systems [2]. IBC is among the two different communication mechanisms that the standard specifies for establishing a wireless body area network (WBAN). Specifically, the IEEE 802.15.6 standard defines the PHY and MAC layers of IBC, which supports quality of service, extremely low power, and data rates up to 10 Mbps. However, IBC in IEEE 802.15.6 does not fully take in consideration some of the practical issues that can be induced from creating a IBC system. Specifically, the IEEE 802.15.6 MAC protocol is simply a Slotted Aloha protocol and the parameters are configured to be static and are defined roughly with minimal consideration on human body characteristics.

While MAC protocol-related research has been active in the wireless domain [3], research for MAC designed specifically for IBC systems is still not a deeply considered topic given that many IBC applications currently focus on a single pair of communication devices, which do not require sophisticated packet scheduling. Even the MAC protocols that are proposed for IBC systems have similar limitations such as the IEEE 802.15.6 MAC [4]. In this work, we see this as a limitation and perform a set of preliminary empirical studies to identify the channel characteristics of an IBC channel, the human body, and reflect these findings to the MAC-layer parameters.

This work proposes IB-MAC, a centralized TDMA-based MAC protocol designed specifically with human-body related parameters in consideration. IB-MAC especially focuses on the transmission latency that the human body introduces as it delivers EM waves through the human body communication medium. We show that the impact of carefully selected guard times, that protect subsequent packet transmissions from colliding, are crucial in maintaining a high goodput performance and managing a low duty cycle (for low-power system design) using an extensive set of simulations. Our results show that maximum achievable goodput of IB-MAC is 452 kbps in a 1 Mbps channel environment, and achieves 9% of radio duty cycle, with a packet delivery latency of ∼18 ms when data slots are fully allocated and the network stabilizes.

Specifically, this work makes the following contributions.

- We perform empirical studies on the performance of electro-magnetic (EM) signal transmissions on the human body. Specifically, we show experimental results on the signal attenuation and transmission latency of EM signals on the human body.
- Based on results obtained from our empirical preliminary studies, we design IB-MAC, a IEEE 802.15.6 PHY-compliant MAC protocol for intra-body communications. The performance of IB-MAC is evaluated through simulations.
- Performance comparisons with standardized and state-of-the-art MAC protocols of IBC systems show that the impact of configuring a proper guard time with respect to empirical transmission latency measurements can have a high impact on IBC systems’ performance.

The remainder of this work is structured as the following. In Section 2, we introduce the concept of IBC and discuss related work in this domain. Section 3 presents our preliminary empirical study on the performance of EM wave signals on the human body communication channel and Section 4 introduces the design of IB-MAC, the main contribution of this work. The performance of IB-MAC along with comparisons with existing protocols are presented in Section 5. We provide a set of discussion points for future research in Section 6 and conclude the work in Section 7.

## 2. Background of Intra-Body Communication and Related Work

We start this work by providing an overview of intra-body communication (IBC) technology and some related work that deals with applications and network protocols in the domain of IBC.

### 2.1. Overview of IBC

In an IBC environment, communication devices are attached directly to the human body and communicate via electrodes exchanging electro-magnetic (EM) or ultrasound signals traversing through (mostly) the skin and muscles. Therefore, a noticeable characteristic of IBC systems is the fact that the human body acts as a common communication medium for all connected devices. EM or ultrasound signals, once transmitted through a device, will propagate throughout the human body for any of the other devices to easily capture. Such a feature suggests that the communication patterns of IBC, although wireless, will be similar to a wired bus communication. Previous approaches using ultrasound for IBC takes on the benefit that the attenuation of the signals spread well throughout the human body compared to the air-medium [5]. However, ultrasound-based IBC systems possess limitations in the amount of data it can transfer due to the propagation latency compared to EM waves. While being suitable for small amounts of transmissions, we find this approach less suitable to apply in various application domains which include entertainment and streaming services [6] that require high amounts of data transfer. Therefore, in this work, we focus on IBC systems that utilize EM waves for communications rather than the ultrasound based approach.

### 2.2. MAC Protocols for IBC

Most applications in the IBC domain are still utilizing small sized messages to enable services in healthcare [7], daily life management [1], or interactive games [8]. However, as the technology matures, IBC applications are targeting to cover a more variety of applications that require high data transmission speeds [6]. Furthermore, the number of devices that utilize the IBC channel will increase. While most of the current applications focus on a pair of devices communicating with each other, an increasing number of IBC devices connected to the human body means that we will potentially need some form of MAC layer to effectively manage their transmissions and increase system-level efficiency. Several previous efforts in the MAC protocol domain has been designed to to serve such applications in IBC envrionments. Phang et al. [9] proposed a wearable health tracker system and tested their TDMA protocol, and Santagati et al. [6] proposed UWB acoustic wave based IBC platform with frequency hopping. However, such approaches have limitations in the fact that they simplified the body channel model by heuristically configuring guard times for body channel propagation for EM waves or acoustic signals. While standards for IBC communication exist (IEEE 802.15.6 [2]), which define PHY and MAC layer specifications, we see limitations in the parameters that these protocols define, given that many of these parameters are taken from the wireless (air medium) channel characteristics.

In this work, we present IB-MAC, a MAC protocol suitable for use in IBC systems. We focus on the EM wave based IBC and also keep in mind the fact that MAC layer parameters need detailed understanding of the human body’s signal propagation characteristics on an empirical perspective. IB-MAC is also designed to suit the high data rate requirements of many applications. In doing so, we first observe in detail how electrodes should be (or can be) connected to the human body communication medium to achieve such goals.

### 2.3. IBC Circuit Coupling

In forming an IBC network, depending on how the electrodes are attached, we can define two different types of communication methods: (1) Galvanic Coupling and (2) Capacitive Coupling. In a capacitively-coupled circuit, the ground electrode is not attached to the body and only the signal electrode is attached. Signal transmission is performed by creating an EM field near the body. The first concept of IBC [1] was that the transceiver shares the earth ground via a capacitive path, which targets delivering small amounts of data between two people. Varga et al. [8] designed an IBC platform for smart environments using 2–8 MHz carrier frequency, conducted several experiments regarding the IBC signal characteristics with various metrics related to measuring signal qualities (e.g., signal strength, SNR), IBC transmission performance, and the usability of the system in multiple use cases by implementing several hardware platforms for IBC systems. The work by Hachisuka et al. [10] used galvanic coupling to find an optimum frequency for the transferring signal through the body. In a galvanically-coupled circuit, the ground electrode is also attached on the body together with the signal electrode. The method is also called a waveguide approach; technically, the human body is used as a waveguide or wire and the ground will be dependent with a transmitter and receiver devices. As a result, in a galvanically-coupled system, the signal flows via inside body and the EM wave will be tied with the human body, not flowing around it.

**Figure 1:**
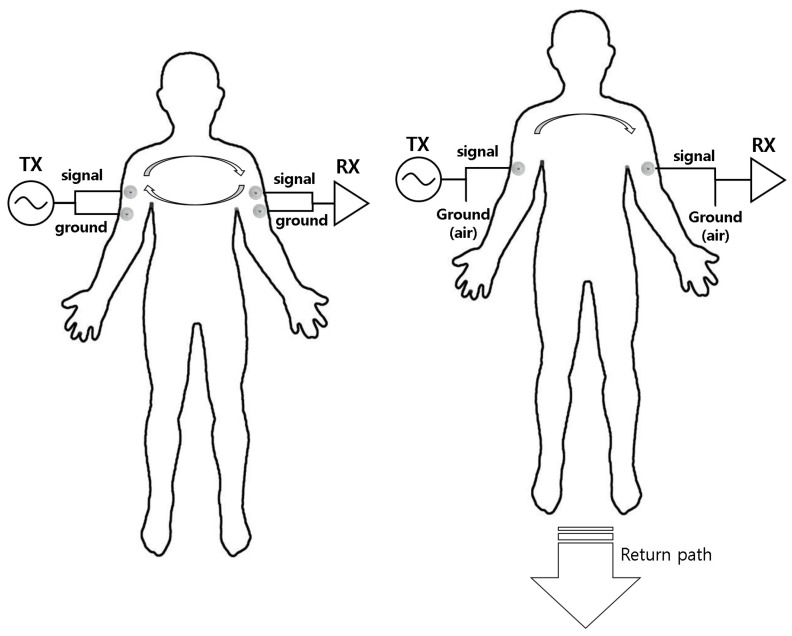
Coupling in an IBC environment.

As we can imply, galvanic coupling tends to have more resistance to external interference factors compared to capacitively coupled circuits, but will require higher energy to transmit its signals. Thus, it will result in a relatively lower data rate [11]. Figure 1 illustrates how the two communication methods differ in terms of electrode connectivity.

Based on such observations, we next present a preliminary study on the empirical studies performed to quantitatively understand how an IBC MAC should be designed with respect to various communication-related performance characteristics.

## 3. Preliminary Study on EM Signals for IBC

The signal attenuation patterns and propagation latency are both important features to understand when designing communication protocols. From the signal attenuation patterns, we can understand how the EM signals flow through the body, and thus observe the potential possibility of exploiting spatial reuse on the common communication medium. Furthermore, the attenuation patterns will show how far the EM signal will travel the body, which will provide additional implications on how the MAC should be designed. By understanding the propagation latency, the MAC protocol can be designed so that it has an understanding of the guard times it must configure to assure safe packet transmissions in an IBC setting. In this section, we present two preliminary experiments we performed to empirically understand the signal attenuation and latency patterns of EM waves.

### 3.1. Signal Attenuation

**Figure 2:**
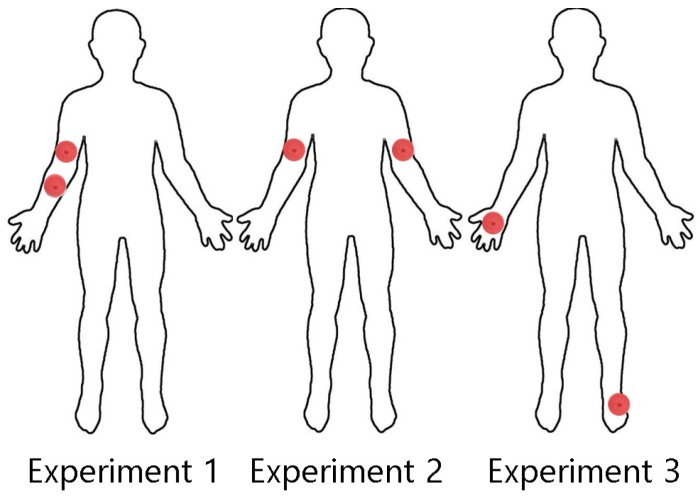
Three different experiment settings for testing the EM wave signal attenuation.

As aforementioned, an IBC circuit model can be designed in two ways, galvanic-coupling and capacitive-coupling. As previous research shows, galvanically-coupled circuits have higher endurance from external interference factors compared to capacitively-coupled circuits. On the signal attenuation perspective, lower attenuation on the human body leads to better communication performance. To validate this, we conducted experiments to compare the performance of the two IBC mechanisms while placing electrodes at the same locations on the body. The location of how electrodes are connected on the body is illustrated in Figure 2, the distance between each electrode is shown in Table 1. For galvanic coupled circuit testing, each red point represents both electrodes of a single device and the capacitive coupling experiment, and the red indicates where the “signal” electrodes are connected with the ground electrode floating.

**Figure 3:**
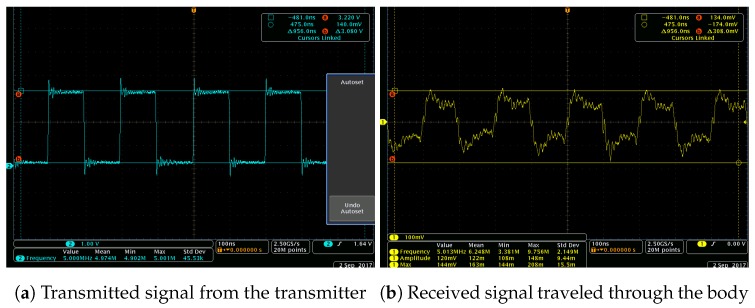
Transmitted and received signal.

Note that the attenuation of a signal is affected by the frequency of the EM waves; therefore, we set the center frequency of the EM signal at 5 MHz, which is reported as the lowest impedance of the human body on the frequency perspective [10]. To generate the EM wave, we implemented a simple program using Atmel SAMR21 [12], which generates 5 MHz base signal output to its GPIO pins. We present the 5 MHz base signal and the generated signal from the GPIO pins in Figure 3a,b. All experiments in this preliminary study was conducted over eight study participants, with an age range of 21–36 and three were female. These participants were especially selected so that different body channel characteristics could be represented (e.g., body fat mass and ratio). We note that the participants were not asked to perceive a specific emotion state nor were they asked to make specific motions during the study. Furthermore, we note that, in all our empirical experiments, the devices are battery powered and the same type of device was used for signal transmission and reception. Given that small differences in the testing environment can introduce deviations in the results [13], we made sure that the testing environment was as stable as possible across different test subjects.

To compute the EM wave signal attenuation on the body, we collect the voltage level data for the input and output signals. All voltage levels were computed as root mean square (RMS) voltage, as in Equation (1). For each $V_{rms}$ sample, we collect 20 M points from an oscilloscope at 2.5 G samples/s, which translates to one $V_{rms}$ sample every 8 ms. Based on this, we compute the attenuation using Equation (2):

(1) $$V_{rms}=\sqrt{\frac{1}{n}\times \left(v_{1}^{2}+v_{2}^{2}+v_{3}^{2}+\ldots +v_{n}^{2}\right)},$$

(2) $$Attenuation=20\times log(\frac{V_{out}}{V_{in}}).$$

**Figure 4:**
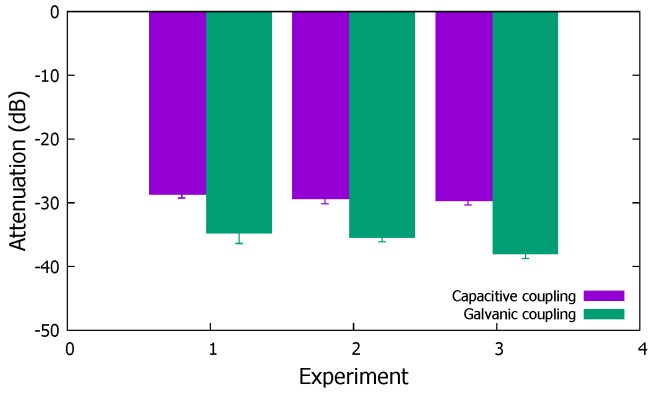
EM Wave attenuation for capacitive and gavanic coupling with different electrode distances.

In terms of EM wave signal attenuation for the three experiments, we can see from Figure 4, where we present the average and standard deviation of link attenuation from our experiments, that the three locations did not show a significant difference. However, we can see here that the capacitive and galvanic coupling cases show a noticeable difference in the signal attenuation. Specifically, while the capacitive coupling shows −27 dB attenuation, that of galvanic coupling was −36 dB. We note that performing experiments on eight people (five independent samples collected for each subject) with different characteristics (e.g., body fat mass) did not have a significant impact on the attenuation patterns in our measurements. As expected, with minimal external interference, the capacitive coupling method shows a much better attenuation performance than gavanically coupled electrodes in IBC. Furthermore, as the results show, when body-attached devices from any location transmits an EM wave, devices attached anywhere on the body can well-receive the EM wave; this is an implication that spatial reuse cannot be effective on the human body channel.

### 3.2. Propagation Latency

**Figure 5:**
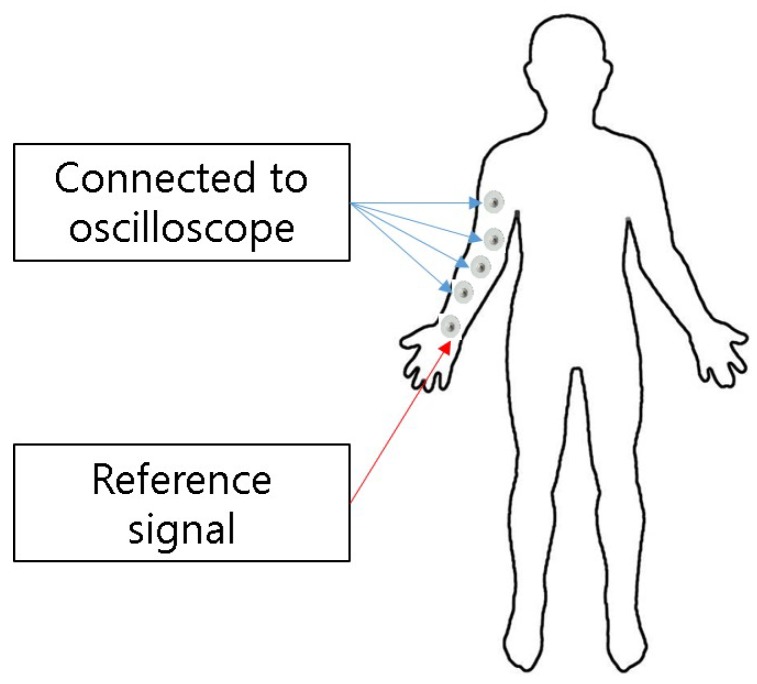
Experiment settings for propagation delay measurements.

Latency computation was done by comparing the peak time stamps for the input and output signals. To compute the EM wave signal’s propagation delay within the human body, we attached electrodes as illustrated in Figure 5. For each experiment, the distance between two electrodes was configured to 10, 20, 30, 40, and 50 cm, respectively. The same eight people that participated in the study performed the latency experiments as well. The latency measurements were made using an oscilloscope connected to both battery powered transmitter and receiver nodes to maintain time synchronization between the two devices.

**Figure 6:**
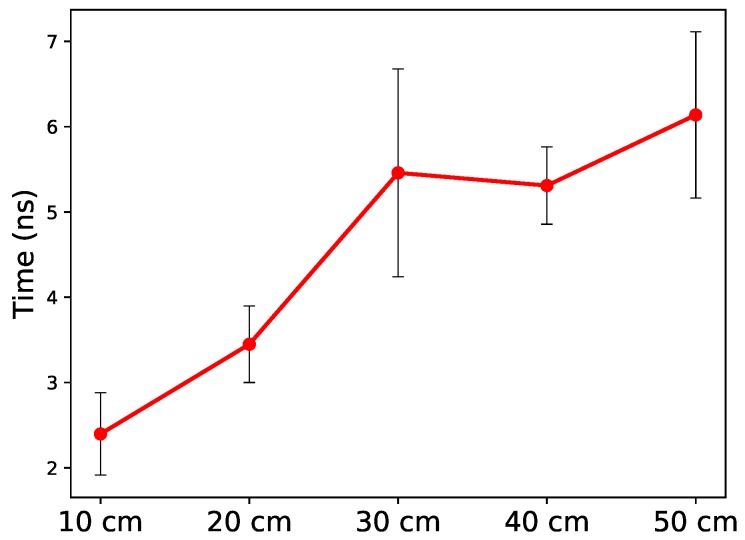
Propagation delay (mean and standard deviation) through the human body for eight study participants.

As Figure 6 shows, where we plot the average and standard deviation of the observed propagation latency, the propagation latency gradually increases with increasing transmission distances. Quantitatively, the propagation delay can be computed as ∼0.94 ns per 10 cm. Indeed, we can notice a large variation in the measurements and we conjecture this to be an effect of small artifacts and blood volume transition in the body and uncontrollable differences between different human bodies.

Given that an electric signal (in the air) travels 30 cm every nanosecond, the propagation latency is approximately 3–4 times slower when the EM waves are transmitted through the human body. This suggests that, when we design time-sensitive protocols for IBC networks, the added latency overhead should be carefully considered.

Finally, we note that the findings above, for both attenuation and propagation delay, are based on an experimental environment using our 5 MHz PHY layer carrier. Observations made using different PHY layer characteristics may show different results. Nevertheless, as our protocol design and evaluations show, this work emphasizes that there should be a careful understanding on the empirical performance characteristics of IBC systems as network layer protocols are designed.

## 4. IB-MAC

Based on the findings from our preliminary study, we design IB-MAC, a centralized TDMA-based MAC protocol for IBC systems. We select to design IB-MAC using a TDMA-based algorithm given that, as we later show, Carrier-sense multiple access with collision avoidance (CSMA/CA)-based approaches poorly perform under the propagation characteristics of a body channel environment. Furthermore, we can say that the attenuation of EM waves in the human body (∼36 dBm) will not affect the communication quality between devices, allowing for configuring a single hop network. Such human body characteristics make it feasible to design a low-complexity TDMA protocol for human body channels. In designing such a MAC protocol, it is important that we well define the structure of the superframe. When doing so, we carefully take in consideration the signal propagation latency induced by the human body. Given that a superframe for a TDMA protocol will include various data types and the interaction of multiple devices, the guard time between such different types of slots becomes an important factor to consider. Note that the guard time configuration depends heavily on the characteristics of the communication medium. For example, IEEE 802.11 a/g/n/ac standard sets the guard interval as 800 ns, or as 400 ns optionally. This adds an 11–20% overhead. In the IEEE 802.15.4 standard, the guard interval is set as 24 μs also adding a 20% overhead. Previous work and standards for IBC, based on such observations, have set the guard time with respect to the propagation speed of EM waves in air medium settings. In the IEEE 802.15.6 standards, for example, the guard interval is set as 85 μs as default plus a small amount of room to account for the clock drift times. Referring to such MAC/PHY layer standards, we can assume that the guard interval is not currently treated as an important feature in the design of an IBC system protocol. However, to design a more effective protocol, we argue that there needs to be a better understanding of the EM wave propagation latency in the human body.

### 4.1. IB-MAC Structure

**Figure 7:**
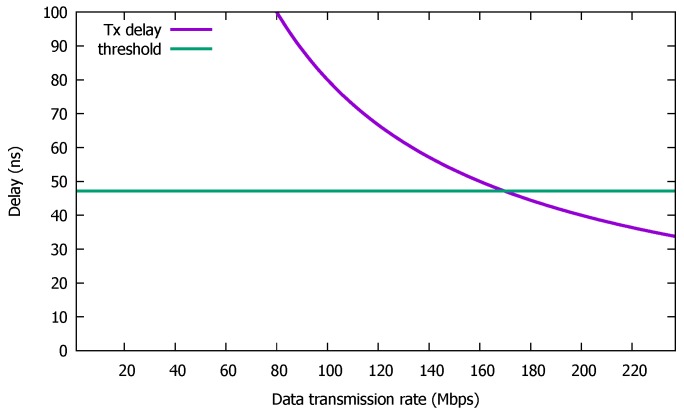
One byte transmission time vs data rate. This delay represents the time that a 8 bit guard time can effectively hold for different data rates.

Time-division-based protocols, such as IB-MAC, should carefully consider how the guard time is set with respect to the transmission latency. To be inserted between each frame, this guard time literally “guards” the packet transmissions within the frame, given that if other transmissions occur before the guard time passes, in a fully connected architecture such as IBC systems (all nodes can hear the transmission of all transmissions in the network due to propagation through the human body), the original packet may collide with the new. In IB-MAC, based on the quantitative results collected in our preliminary studies, we set 1 byte (8 bits) of guard time for each packet transmission slot. This is made under the assumption that the maximum distance that can be achieved on a typical human body is 2.5 m (finger to toe) and we set the guard time to be at least two times of this latency with respect to the EM wave’s propagation speed (i.e., ∼10.6 cm/ns)—a typical transmission range for IBC is 2 m, but 5 m for some extreme cases [14]. Since the transmission delay (i.e., guard time) will be considered within the packet structure, in which we configure byte-level arrangements to account for any synchronization issues, we fix a guard time of 1 byte—8 bits. This time, while not significant, is theoretically sufficient enough to support a data rate of 170 Mbps in the IBC channel. Figure 7, in which we plot the threshold of single byte transmissions with respect to varying data rates, shows that having a single byte of guard time will hold effective until exceeding a data rate of 170 Mbps. IB-MAC is originally designed to operate at 1 Mbps; thus, this is a sufficient amount of time to protect the packets. Further optimization on the guard time length can be made, but we pad additional bits to match the 1 byte size to accommodate for potential clock drift as well.

Given the 1 Mbps physical layer data rate, the guard interval of IB-MAC would be 8 μs. Furthermore, with the characteristics of human body connections in mind, all nodes in the network can be reached from the coordinator node via single hop. In addition to these factors, we design the protocol with the following design goals:

- Given the increasing trend in wearable devices, we target designing a MAC protocol that supports more than 20 nodes, 100 at the maximum.
- Based on requirements of healthcare and military applications, we set the maximum size of a data frame to 50 bytes. For larger packets, the MAC protocol should be flexible enough to account for more data slots to a single transmitter node.
- There should be a way of prioritizing packets that need to be delivered urgently and nodes should be able to join the network with minimal latency.
- The length of the superframe, which determines the frequency of packet transmissions should be kept small to achieve high throughput and application-level message delivering latency.

**Figure 8:**
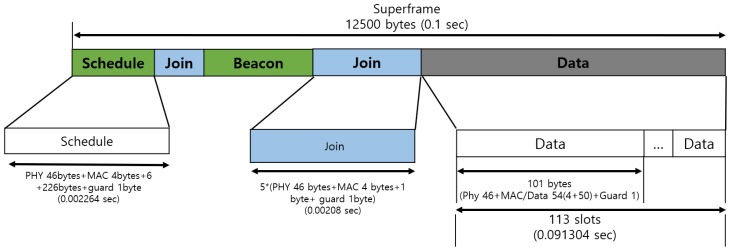
IB-MAC superframe structure.

Based on such design goals in mind, we present the superframe architecture of IB-MAC in Figure 8. The total size of a single superframe is set to 12.5 Kbytes and the superframe includes three types of fields: (1) Schedule, (2) join, and (3) data. We also save one reserved byte for future use.

The schedule field is used via downwards communications (from the coordinator to individual nodes) and is used to disseminate the transmission schedules for all nodes in the network. This field also implicitly serves the purpose of network-wide time synchronization. The join field in the superframe is used via upwards communications (from individual nodes to the coordinator) to deliver join request messages of individual nodes. New nodes can participate in this phase or existing nodes that wish to change their data transmission rate can also send packets in this phase. The join phase is the only period in IB-MAC’s superframe in which nodes compete to access the medium; thus, this is a contention access period based on CSMA/CA. Once the nodes send join messages, the following second round of schedule field responds to these requests, so that newly joining nodes need not wait an entire superframe period to send their data. Given that one of our design goals is to minimize the join latency, we find this an important aspect of our system. Nodes that did not join the system prior to this second schedule distribution phase can access the system using the second join period, but, in this case, they are asked to wait for the data transmission field/phase to end, prior to receiving a schedule for the TDMA network. Finally, the data field is divided in 113 data transmission slots, with guard times, to allow for up to 100 nodes (additional slots for nodes with more than one slot requirement), while assuring a minimal data rate of 300 kbps to support the applications such as video streaming to mixed reality headset devices, standard definition (SD) quality video streaming can be supported.

### 4.2. Frame and Packet Format

**Figure 9:**
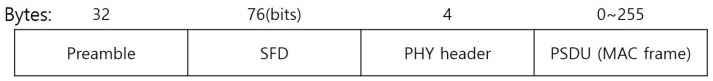
PHY frame format.

All IB-MAC packets are encapsulated in a PHY frame. We design the PHY frame format to meet the requirements of the IEEE 802.15.6 human body transmission standards [2]. As Figure 9 shows, the size of a PHY frame is 46 bytes. The only change that we make to the original standards is the repetition of the preamble and start-frame-delimeter (SFD). According to the IEEE 802.15.6 standards, this preamble and SFD should be repeated four to eight times, but we reduce this to once in our design. As a result, each packet starts with 32 bytes of preamble, 76 bits (9.5 bytes) of SFD, 4 bytes of PHY header, and the PLCP (Physical Layer Convergence Procedure) Protocol Data Unit (PPDU) (e.g., MAC frame) follows. While this length is 45.5 bytes, we put a 4 bit padding to the header to create a 46 bytes length.

**Figure 10:**
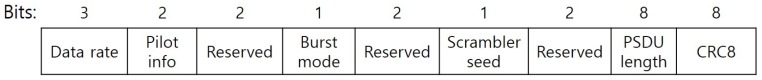
PHY Header format.

The PHY header, as illustrated in Figure 10, is designed to be identical to the IEEE 802.15.6 standards. IB-MAC, while configurable, sets the Data Rate filed to 011 (i.e., 1 Mbps) and Pilot Info field to 00 since this is not used in IB-MAC. Other fields in the header, except for the PSDU length field, which is configured with respect to the packet length, is set as the standard’s default.

**Figure 11:**
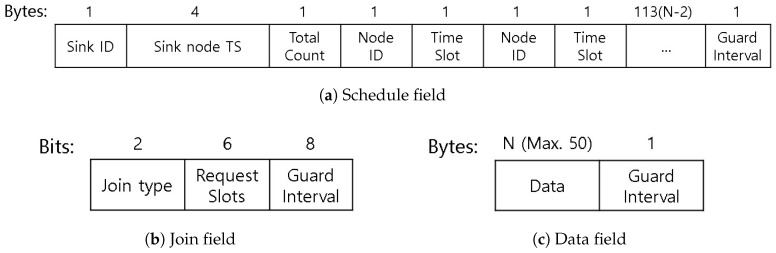
MAC frame format.

The MAC layer frames consist of the last parts of the PHY frame. Figure 11 presents the three different types of MAC frames as briefly mentioned above. The packets used in the schedule field are presented in Figure 11a. The sink ID and time stamp are shared for network-wide time synchronization. In addition to this, the total number of nodes in the network and information on the data transmission time slot allocated for each node is included in this large sized packet. Through this schedule packet, we can exchange the schedule for a maximum of 113 nodes in the network by sending packets at all available time slots. This slot count was select based on our application level requirements of supporting up to than 100 nodes in the worst case, with some room to fill up the superframe with effective slots for nodes that require more than one slot for transmissions. Finally, the guard interval protects the schedule packet from the join packets that follow in the superframe architecture. Note that, on the second schedule field of the superframe architecture, the exact same frame structure is transmitted. We agree that, if no packets join, this can be a waste of resources, but we see the importance of fast node network joining as an important feature and allow for such inefficiencies. As part of future work, we plan to investigate into the use of a dynamic superframe architectures that utilizes such additional information. The one reserved byte in IB-MAC can be used to identify such situations and adjust IB-MAC to be applicable in such cases.

The join packet’s format in Figure 11b is 2 bytes in length. The first two bits include the join type, indicating new join, detach, and modify rate cases. Nodes can request up to 64 slots and we set 1 byte of guard time. Note that the size of the join packets are much smaller than the schedule packets. As aforementioned, the join phase is a contention access period, where many nodes compete for time slots to reach the coordinator node. Hence, it is important that packet sizes are kept small to minimize the chances of packet collision and contention.

Finally, the data packets (Figure 11c) are simple and just include the data to be transmitted to the target destination with a guard interval at the end. The maximum data transmission size is set to 50 bytes in IB-MAC based on its design requirements. Note that nodes that require transmitting more than 50 bytes of payload can compete again in the join period to be allocated additional data slots.

**Figure 12:**
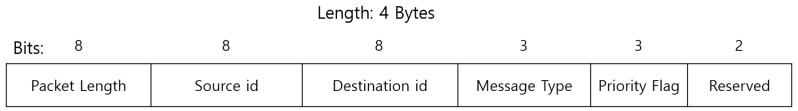
MAC header format.

The three types of packets discussed above are actually the payload of the MAC header. The MAC header precedes the three packet types and the design of the MAC header in IB-MAC is presented in Figure 12. Specifically, the MAC header has a size of four bytes. The packet length denotes the length of the payload in the MAC packet, and the message type field specifies which of the three MAC packet formats are used. The source and destination fields are designed to be 8 bits each given that the goal of our scheme is to support >100 nodes. With these 8 bits, we can potentially represent node IDs from 0–255 (255 is reserved from broadcast messages). The priority flag is used so that, if used, the schedule coordinator can refer to this information to prioritize the requests from specific nodes in the network. This priority field can be used to represent different levels of message priorities as the IEEE 802.15.6 standard specifies.

### 4.3. IB-MAC Procedure

**Figure 13:**
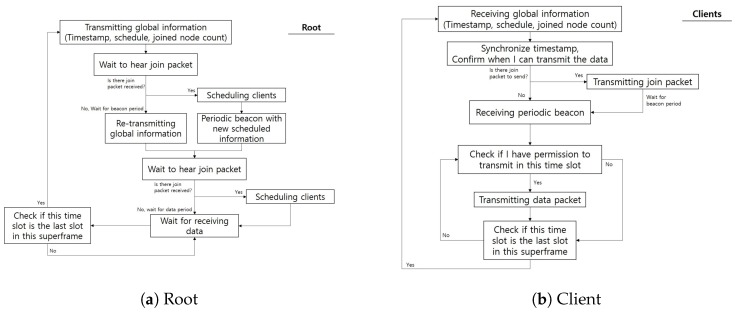
IB-MAC state diagram for root and client devices.

We now present the operations of the IB-MAC when functioning. When a superframe period starts, the schedule information is transmitted to all nodes by the coordinator/sink node in the body area network. All nodes that are already participating in the network will have information on which data slot to use to transmit their data. During this phase, the individual nodes synchronize their timers with respect to the coordinator node’s timestamp. From the join phase, new nodes can send join packets and existing nodes can adjust their transmission rates (e.g., request additional data slots). Again, all nodes may compete for the channel access in this period; thus, IB-MAC introduces a random backoff-based contention avoidance scheme during this phase. During each join period, a maximum of five nodes can join the network. This means that, at each superframe, ideally, 10 nodes can join the IB-MAC-based network. When the superframe enters the data transmission phase, all individual nodes will count the current slot number to check its transmission slot. This can be easily done, given that the node frequently synchronize their packets with the coordinator. The operations discussed here are summarized in Figure 13.

In terms of radio duty-cycling, all nodes must be awake and listen to incoming packets in the two schedule phases of the superframe. If the node has already joined the network, there is no need for the node to keep its radio on during the two join periods. Only the nodes that desire to join the network or make changes will keep their radios on at this time. In the data exchange period, only the nodes that need to send/receive packets need to be awake during a data slot. If we assume that all transmissions are upwards (from individual nodes to the coordinator) only, the transmitter and coordinator pair need to keep their radios on during a single data transmission slot. It is true that IB-MAC requires the coordinator node to continuously turn on its radio, but we allow for aggressive duty cycling at the individual nodes. We take this design choice given that, in many cases, there is a gateway node that interconnects the BAN nodes to a larger Internet architecture and this device typically possess more energy resources compared to the other leaf nodes.

## 5. Evaluation

We now evaluate the performance of IB-MAC by comparing the performance of the IEEE 802.15.6 standards-based CSMA/CA and Slotted Aloha MAC protocols. To evaluate the performance of IB-MAC, we implemented an event-based MAC protocol simulator for each of the three protocols using Java. Specifically, the simulator is designed so that nodes can be positioned with different link level loss rates and can be configured to generate a given amount of (randomized) network traffic. We will specify the parameters used in our simulations below. Through the evaluations, we target comparing the goodput performance and duty cycling performance under various experimental conditions.

**Figure 14:**
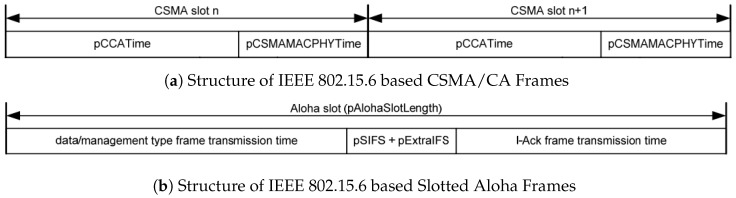
Structure of IEEE 802.15.6 based CSMA/CA and Alotted Aloha frames [2].

For IEEE 802.15.6 based CSMA/CA MAC, since the standard allows only specifies the Slotted Aloha approach for human body communication, parameters in designing the MAC protocol was taken from the RF communication-based portions in the IEEE 802.15.6 standard. For both CSMA/CA and Slotted Aloha, by comparing various parameter settings, we select the best parameters and optimize them to meet the best-possible goodput performance. Furthermore, since our proposed protocol mostly follows the PHY layer of the IEEE 802.15.6 standard, all frames of the three protocols use the same PHY header structure. Specifically, the PHY layer for all three experiment cases use the PHY header discussed in Section 4.2. Each MAC layer implementation parameters are decided as Table 2. The guard interval of the standard was set to default, 85 μs. The allocation slot of CSMA/CA and Slotted Aloha is illustrated in Figure 14a,b, which we have taken from the standard documentation. Regarding the probability or contention window size for transmission slot allocation, we followed the user priority (UP) specifications as defined in the IEEE 802.15.6 standard. Each probability and contention window size is presented in Table 3.

**Figure 15:**
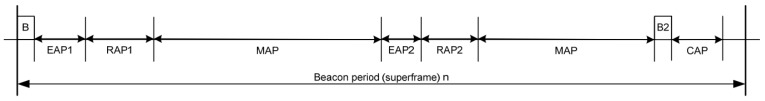
Beacon based IEEE 802.15.6 superframe [2].

Note once again that we tried to maximize the performance of both standard-based MAC protocols, so we assume that all nodes which can be reached in the network have already joined, so that there are no other nodes that need to newly join in the network, and the slot allocation field (i.e., RAP1) fills the overall superframe (as denoted in Figure 15) without any additional beacon period, and we set the MAC payload size to its maximum of 255 bytes.

In addition to comparing with the two standardized approaches, we introduce an additional comparison with a more recently proposed state-of-the-art scheme proposed by Phang et al. [9]. This scheme, as briefly mentioned in Section 2, is a recently proposed TDMA-based MAC protocol for IBC systems. The goal of Phang et al. is to configure a variable sized superframe structure, by adding in new beacon-based sub-superframes in units of 8 data transmission slots. For this work, we configure the PHY layer of this Phang et al.’s protocol to be the same as the other MAC protocols of our interest to perform fair comparisons. A summary of the parameters used are presented in Table 2.

Regarding the network’s experimental scenario, for all experiments, we configure a network of 20 nodes, in which all 20 nodes continuously request for additional data slots. One data slot is requested at each superframe interval for the CSMA/CA, Slotted Aloha [2] and Phang et al. [9], and for IB-MAC, nodes will freely compete for the 10 join slots available at each superframe. All 20 nodes are set to be connected via single hop to the coordinator node and unless separately specified, we configure no link loss on the body channel environment.

### 5.1. Goodput

We first start by comparing the goodput performance of each protocol to show whether IB-MAC shows comparable goodput performance with other protocols (IEEE 802.15.6 based Slotted Aloha and CSMA/CA, state-of-the-art TDMA MAC protocol for IBC [9]) and also to see if it can satisfy the high data rate requirements of demanding applications. Based on the MAC parameters discussed in Table 2, IEEE 802.15.6 based CSMA/CA can potentially achieve a goodput of up to ∼600 kbps, and Slotted Aloha can achieve up to ∼330 kbps. While these numbers are for the extreme optimal cases, we can see a big gap between the two MAC protocols. This is mainly because in Slotted Aloha, each slot length (based on the standard) should be set as two-times of the packet transmission delay, consisting of the data transmission, guard interval, and acknowledgement periods. IB-MAC, on the other hand, guarantees a maximum of 452 kbps of goodput based on optimal analysis.

**Figure 16:**
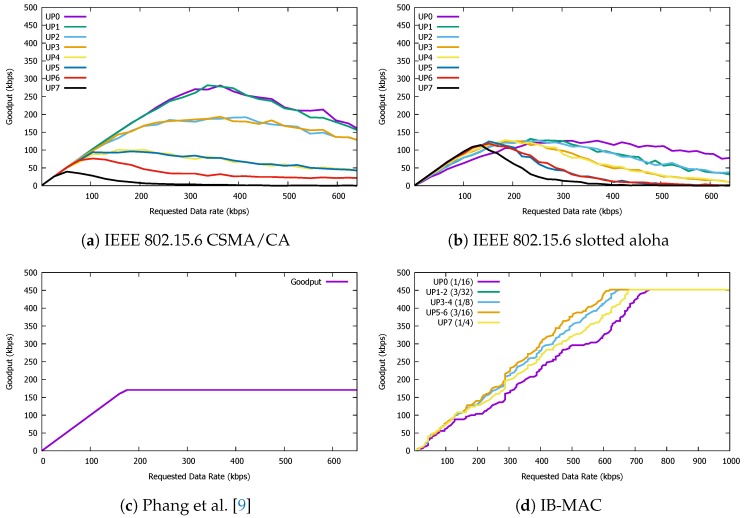
Goodput comparison for IB-MAC and compared schemes.

While the above values are for the optimal scenario, we now present results from a simulation study on the performance of the three MAC protocols of our interest. The simulator is customly designed as an event-based simulator implemented in Java. We take the parameters discussed in Table 2 for the implementation of each MAC protocol in our simulator. Furthermore, the data transmission probabilities within a superframe operation is determined using the standard specifications as in Table 3. In Figure 16, we present the goodput performance achieved by each of the MAC protocols for varying amounts of data requests at the individual devices. Once again, we devise a network in which up to 20 nodes request to send data to a central node in the IBC-based body area network. As the *x*-axis increases, nodes in the network ask for more data transmission slots, specifically, one additional data slot is requested on a per-superframe basis. The only exception here is that in IB-MAC, since more than one node can send join messages in the join period, in the ideal case, up to 10 slots can be allocated at the same time, but, as mentioned, this occurs on a contention basis. All nodes in the network uniformly ask for the same number of data slots and we assume that all 20 nodes are physically an equal distance away from the coordinator nodes. All transmissions at a data slot are for the maximum size of the data transmission slot (maximum payload length). Results in Figure 16 shows that, while the protocols exhibit slightly different behaviors for varying user priority (UP) values, IB-MAC shows the best goodput performance. For IB-MAC, even when the network requests for more than 420 kbps, the goodput does not stabilize until nodes can successfully join the superframe schedule. This is an effect of the contention-based join phases of our superframe. Nevertheless, notice that the CSMA/CA-based scheme shows a goodput decay after requesting 350 bits per second (bps) even in the best case. The best case performance for CSMA/CA is ∼300 kbps. Similar decays can be observed from Slotted Aloha as well. We denote that such performance decay with increasing traffic request is due to the congestion that these protocols introduce to the network. On the other hand, while IB-MAC also shows a fluctuating performance at the beginning of the network setup phase (mainly due to the effect of nodes participating in contention in the “join phase”), once the slots are all allocated to nodes, the network good put stabilizes. It is true that new nodes (or existing nodes) may request for additional slots, but, if the slot allocation is full, then the coordinator node in IB-MAC will disregard these join requests to serve the existing nodes’ traffic. Note that the detailed parameters used for CSMA/CA and Slotted Aloha are for the best performance parameters based on empirical studies prior to collecting the simulation data for these graphs.

When comparing the performance with Phang et al. [9], we can see that IB-MAC saturates at a much higher goodput. This is because the performance of Phang et al. saturates at ∼175 kbps, due to the variable structure of their superframe architecture; if more slots are requested, the superframe length simply increases. Therefore, the amount of data sent within a given time frame does not change dramatically. Furthermore, given a short data slot of 16 bytes, a guard time of 125 μs can be considered as too much overhead, and this limits the maximum goodput of the protocol. We point out that the performance observed from our simulations match that of what is reported by Phang et al. in their work; thus, this serves as evidence that our custom simulator performs properly and is valid to use in our experiments as well. In contrast to this long guard time, IB-MAC, based on empirical studies, configures the guard time to be equal to the transmission time of 1 byte. With the 1 Mbps data rate that we use in our evaluations, this translates to 8 μs, which is much smaller than the guard time of other compared MAC protocols. Note that the work by Phang et al. [9] does not include any contention access period, and assumes that all nodes have already “joined” the body area network. Therefore, there is no performance difference for different user priority values (this is why this figure consists of a single line plot).

### 5.2. Duty Cycle

Given that one of our main goals is to maintain a low power profile for the IBC nodes, it is important that we can save energy resources based on turning the radio component on and off. Therefore, the duty cycle of the radio has a direct impact on the lifetime of the body area network system. Therefore, we now present the radio duty cycle that each of the three MAC protocols can achieve. Again, the parameters and scenario of slot allocation is kept the same as the previous goodput experiment. We also make sure that the length of different slot types in the IEEE 802.15.6 MAC protocols do not affect the radio duty cycle.

**Figure 17:**
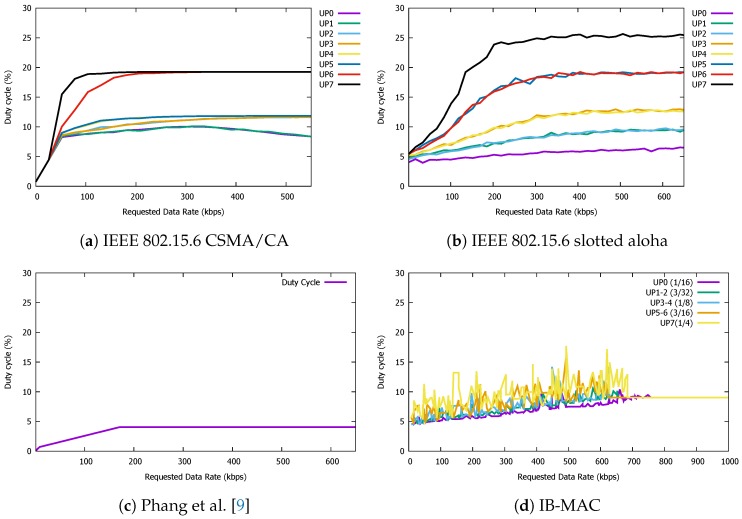
Duty cycle performance of IB-MAC and compared protocols.

In Figure 17, we plot the radio duty cyle for our three MAC protocols of interest. On the *x*-axis, we increase the superframe count sequentially, which means that one superframe period passes for each *x*-axis tick; thus, a new node joins if possible. For IB-MAC, we increase the superframe count until all the frames are full and the duty cycle saturates. Once IB-MAC stabilizes, we observe a duty cycle of ∼9% for all UP conditions. However, the CSMA/CA and Slotted Aloha methods show more variance with varying UPs. Quantitatively, for CSMA/CA, while the duty cycle stays low with low UPs, since nodes more actively compete for the channel at high UPs, the duty cycles accordingly increase as well. The same trend holds for Slotted Aloha as well for the same reasons.

While, in some cases, the radio duty cycle is lower for CSMA/CA and Slotted Aloha compared to IB-MAC, if we compare for the same goodput levels, we argue that IB-MAC shows a competitive performance. The relatively high duty cycle of IB-MAC is mainly due to the long wakeup times in exchanging the schedules in the beacon periods. This number can be further optimized and adjusted by exchanging only the schedule that change and by supporting dynamic superframe sizes. We plan to consider these issues as part of our future work.

The performance of Phang et al. [9] as presented in Figure 17c shows that the duty cycle saturates at ∼4% with increasing requested data rates. Comparing this with that of IB-MAC, we can notice that the saturation point for Phang et al. is lower than IB-MAC to show a more energy efficient performance. However, when combining this result with the goodput plots in Figure 16, we can see that IB-MAC, compared to a recently proposed state-of-the-art protocol, achieves a much higher goodput and is more suitable for IBC system applications that support high data rates. Therefore, when supporting such high demands, the duty cyle performance is sacrificed. A closer look at Figure 17 suggests that, at similar requested data rates up to ∼175 kbps, the duty cycle performance of IB-MAC is similar to that of Phang et al. [9]

### 5.3. Latency

Next, we observe the observed latency of packet transmissions are for the four different protocols of our interest. We define the latency of a successfully delivered packet as the time between the initial intent to send a packet at the individual transmitters, until the receiver node receives the packet. Specifically, for superframe-based protocols, we assume that the packet transmission intentions for all participating nodes (if any) take place at the beginning of the superframe. Note that we compute the latency for only the packets that are delivered to the destination. In other words, the packets that were not assigned a data slot are discarded from the latency computation.

**Figure 18:**
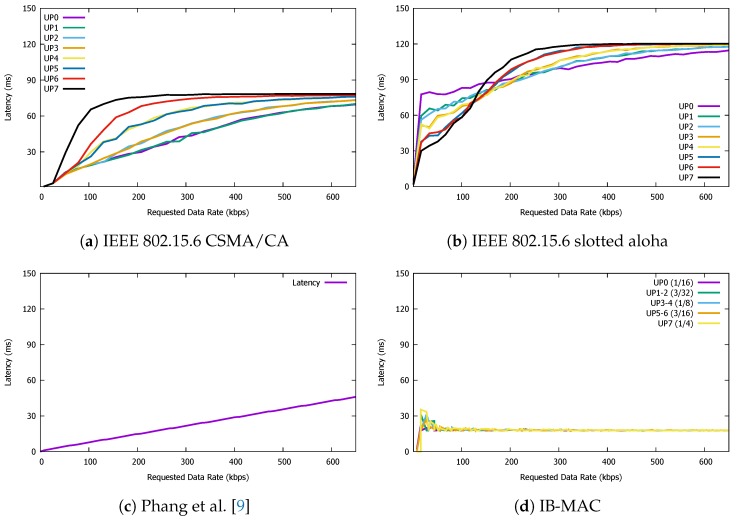
Latency performance of IB-MAC and compared protocols.

The latency results presented in Figure 18 shows that IB-MAC shows a saturating latency performance at ∼25 ms, with the two IEEE 802.15.6 standards saturating at higher values. For Phang et al. [9], the latency performance shows a linearly increasing trend. Since the size of the superframe is proportional to the requested data slots, more data slot requests will lead to long superframe lengths. Naturally, on a single node’s perspective, its opportunity to send its next packet will be delayed as the requested data slots in the network increases.

### 5.4. Impact of Packet Loss

Finally, we examine the impact of packet loss on the goodput performance. For this experiment, we set the channel to introduce a random packet error rate (PER) of 2, 4, 6, 8, and 10%. Under such lossy networks, we examine how the goodput performance of each MAC protocol is impacted. For fair goodput comparisons, all four MAC protocols are configured so that retransmissions due to packet loss are suppressed.

**Figure 19:**
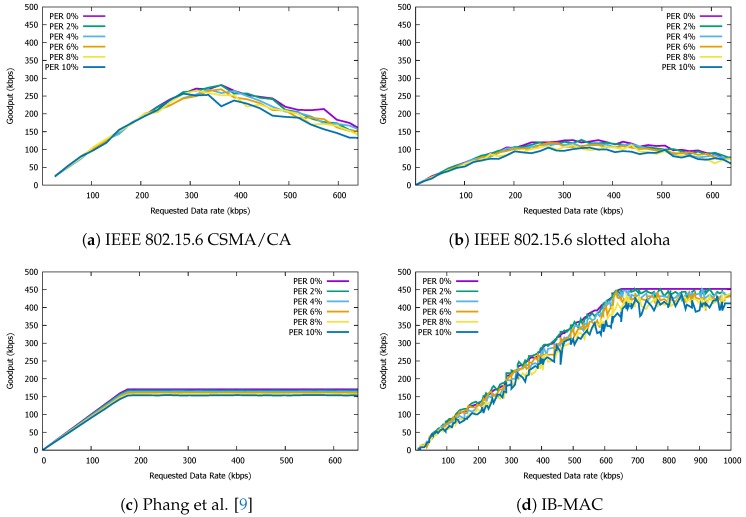
Goodput performance of IB-MAC and compared protocols under various packet error rates.

As Figure 19 shows, all protocols are almost equally impacted by the changes in PER. For the IEEE 802.15.6 standard protocols, we set the user priority to UP0, which showed the best performance under lossy channel conditions. For IB-MAC, we present results for the experiments using UP3, which shows its best performance as well. Note from the IB-MAC goodput performance plots that introducing channel loss leads to continuous goodput fluctuation and longer time to saturate at its maximum goodput. As an extreme comparison, for Phang et al., there is close to no fluctuation in goodput. This is mainly because link losses lead to less chances of join packets successfully making it to the coordinator node, which results in network instability in IB-MAC. Since there is no explicit contention-based access period in Phang et al., the network is fairly stable despite some random packet losses. Nevertheless, we conjecture that a channel loss of 10% will not be common for short IBC packet transmissions given that IBC systems share many similarities with wired communications, in which only small levels of link loss can be observed. Despite the goodput fluctuation, the goodput performance of IB-MAC still outperforms that of other compared MAC protocols.

## 6. Discussion and Future Research Directions

We now raise a few interesting discussion points related to our study and present future research directions that we see important on MAC and networking protocols for IBC systems.

### 6.1. Personalization

Unlike the wireless medium, where the channel characteristics in idle environments are similar and can be generalized, human body characteristics can vary over different people. While some efforts have been made to generalize the human body communication model [15,16,17], there has also been many studies suggesting that a wide range of variability exists as people have different physical characteristic which impacts the communication quality such as impedance and optimal signal propagation frequency [18,19,20]. Furthermore, findings from previous work suggest that the experimental settings can also have an impact on the performance of IBC systems [13]. While we identified only minimal changes on a per-person basis ourselves, our study pool was limited to eight people and with a single type of signal configuration. Unfortunately, there were practical limitations ourselves in capturing data from many study participants; thus, we see a large scale user study to measure the communication performance variations under diverse human samples, and various EM wave configurations to be important before IBC systems can be practically deployed and utilized.

### 6.2. Real-World Deployments

Tied in with the previous discussion points, we find it important to perform large scale pilot studies to understand and design protocols that are suitable for everyday use. Specifically, we need a better understanding of how a person’s motions can practically impact an IBC system’s performance in an empirical way. This work offers some level of empirical study of the EM waves in a human body, but the motions that people make during their everyday activities can form an additional layer of complexity in network/communications protocol design. Again, this is something that we have not considered in the scope of this work, but is an essential step towards realizing practical IBC systems.

### 6.3. Application Requirements

The concept of wireless body area networks have introduced a variety of applications [21,22,23,24]. These applications, however, are so diverse that their application requirements differ quite a bit. Considering that the manufacturing costs are a crucial part of technology realization, we foresee the need for a comprehensive study that summarizes the role of IBC systems in wireless body area networked applications, and the application requirements that different application categories introduce. This work is focused on high data rate applications, as this domain has been seldomly studied until now, but a summary of different application requirements can reveal new design paradigms for IBC systems in general.

## 7. Conclusions

In this work, we designed a transmission latency-aware TDMA-based MAC protocol, IB-MAC, for intra-body communication environments. The design of IB-MAC is based on findings gathered from an empirical study conducted to capture the electromagmetic wave propagation patterns on a real human body, and we perform a set of simulations to validate the performance of IB-MAC compared to the body channel MAC protocols standardized by IEEE 802.15.6 and a state-of-the-art MAC protocol designed explicitly for intra-body communication networks. Our results suggest that the goodput performance of IB-MAC outperforms other compared protocols by as much as three-fold, while exhibiting comparable radio duty cycling performance. We also identify future directions of research for realizing IBC systems in real world applications. While, in its early stages, we envision that the human body communication channel can be an effective substitute for many RF-based wireless protocols that we use today as the number of devices increase, we hope that the findings in this work can initiate many research work in this domain.

## Figures and Tables

**Table 1:** Distance between the transmitting and receiving electrodes in Figure 2.

Distance 1	Distance 2	Distance 3
18 cm	76 cm	209 cm

**Table 2:** MAC parameters in 802.15.6 based CSMA/CA, Slotted Aloha, a state-of-the-art IBC MAC protocol [9], and IB-MAC in our simulations. All MAC protocols operate on a 1 Mbps data rate environment.

	CSMA/CA	Slotted Aloha	Phang et al. [9]	IB-MAC
Allocation slot period	467 μs	6.01 ms	653 μs	808 μs
Number of slots	167	20	Variable with data request	113
MAC payload length (bytes)	255	255	16	50
Guard interval (default)	85 μs	85 μs	125 μs	1 byte
Transmission delay	2.48 ms	2.48 ms	653 μs	808 μs
Acknowledgement period	55 μs	55 μs	no-ack	no-ack
Beacon length	597 μs	597 μs	maximum 653 μs	4.528 ms
Superframe length	78.586 ms	121 ms	5.88 ms × data slot request/8	100 ms
Maximum retransmissions	2 (UP < 5), else 4	2 (UP < 5), else 4	none	none

**Table 3:** Probability and contention window size depending on user priority.

UP	CSMA/CA		Slotted Aloha		IB-MAC
	CWMIN	CWMAX	CPMax	CPmin	CPmin
User Priority0	16	64	1/8	1/16	1/16
User Priority1	16	32	1/8	3/32	3/32
User Priority2	8	32	1/4	3/32	3/32
User Priority3	8	16	1/4	1/8	1/8
User Priority4	4	16	3/8	1/8	1/8
User Priority5	4	8	3/8	3/16	3/16
User Priority6	2	8	1/2	3/16	3/16
User Priority7	1	4	1	1/4	1/4
